# On Fruits and Fungi: A Risk of Antifungal Usage in Food Storage and Distribution in Driving Drug Resistance in Candida auris

**DOI:** 10.1128/mbio.00739-22

**Published:** 2022-05-16

**Authors:** Vikas Yadav, Joseph Heitman

**Affiliations:** a Department of Molecular Genetics and Microbiology, Duke University Medical Center, Durham, North Carolina, USA

**Keywords:** *Candida auris*, antifungal resistance, drug resistance evolution

## Abstract

The continuous emergence of antifungal drug resistance is a mounting concern for the treatment of fungal infections worldwide. While many pathogenic fungi exhibit some level of antifungal drug resistance, the identification of Candida auris has brought this phenomenon to the fore in recent years. C. auris exhibits resistance to all antifungal drugs used for treatment, and it does so at a very high rate, with more than 90% of isolates being resistant to at least one drug and roughly 4% being panresistant. However, the environmental factors driving this exceptionally high antifungal drug resistance remain unidentified. The presence of C. auris on stored apples that are treated with antifungals during storage suggests a possible route to selection of drug-resistant C. auris isolates that may have contributed to the evolution of this deadly pathogen. This study further suggests that the adage “an apple a day keeps the doctor away” may need to be revisited in light of the discovery of C. auris on the surface of apples.

## COMMENTARY

In the study by Yadav et al. (1), the investigators screened fruits for the presence of fungi and identified a large number of fungal species present on the surfaces of fruit. A total of 84 fruits from 9 different plant species were screened from northern India, and a total of 144 yeast strains were isolated, including pathogenic and nonpathogenic species. Among the 84 fruits, 62 were apples (20 fresh and 42 stored), and the investigators then focused on apples because they were the main reservoirs of several ascomycetous *Candida* species. *Candida* strains were isolated from apples that were stored but not from those freshly harvested from orchards. Many isolates were observed to be resistant to agricultural antifungal drugs present on fruit and showed cross-resistance to clinical antifungal drugs. The study then focused on Candida auris isolates that were subjected to whole-genome sequencing for detailed characterization and to understand the basis of antifungal drug resistance.

The *Candida* pathogenic clade of species includes multiple well-known human-pathogenic fungi that are generally present as commensals, including Candida albicans, Candida tropicalis, and Candida parapsilosis among others. These fungal pathogens can cause infections in both immunocompetent and immunocompromised patients and are well known to develop antifungal drug resistance both *in vitro* and *in vivo*. C. auris is the latest addition to this group and was identified in Japan in 2009 (2). Since then, the fungus has been isolated on every continent except Antarctica and was recently highlighted as one of the major emerging fungal pathogens by the Centers for Disease Control and Prevention (CDC). One main characteristic of this species is a high prevalence of antifungal drug resistance, making it a significant concern to public health (3). Until recently, all C. auris isolates have been from patients. The first environmental source for this fungus was identified when researchers isolated C. auris from the sandy beaches in the Andaman and Nicobar Islands, India (4). Based on this discovery, the sandy beaches and marsh regions were proposed to be natural niches for C. auris. While it has been proposed that global warming and climate change could be one factor driving the evolution of this pathogenic fungus, the ecological niches and distribution modes for C. auris remain largely unknown (5).

The investigators of this study screened a large number of tropical and temperate fruits to explore the mycoflora. They focused on apples because they are processed for long-term storage and are available year-long in the market. Interestingly, all of the apples from which C. auris was isolated had been processed and stored in a cold storage facility before marketing. When freshly harvested apples from orchards were analyzed, only one colony of C. albicans was isolated and no other *Candida* species was detected. This observation hints toward the presence/spread of *Candida* species in conditions/places where apples are stored and could lead to changes in fruit mycoflora. However, the fungi were isolated from the surface of fruit and not from the inside, suggesting that fungi were likely transferred to the surface of apples during transport and handling.

*Candida* species are resident within the skin microbiome, and it is possible that *Candida* identified on fruit was transferred via human handling. Starting from picking an apple to its processing, storage, and distribution, almost all of this handling is manual, facilitating potential transfer of fungi from the human hand to the surface of the fruit. That commensal yeasts were obtained mainly from stored apples supports the idea that humans could be substantial reservoirs for these fungi and fruit may be one mode of spread. Thus, it will be important to know at what step these fungi were introduced onto apples. Because humans are involved at every step, one aspect for future studies should focus on testing fruit storage facility workers for the presence of C. auris. Many processing steps between harvesting and fruit consumption provide a hospitable environment for fungal growth and survival. Except for cold storage that takes place at near-freezing temperatures, transport and marketing provide favorable temperature and humidity. Furthermore, the place of marketing is generally crowded and apples are kept in open areas, frequently on roadside trolleys. Genetic analysis of isolated C. auris strains revealed the presence of highly similar strains on different apples as well as different strains on the same apple. This again could be explained by apple storage/distribution at a common facility. The presence of different C. auris strains on the same apple could be due to more local distribution and might indicate a more recent transfer onto apples after release from a common facility. Overall, these results suggest that fruit storage and distribution is a facile mode via which these fungi may spread. Provided that fruit is transferred over a large distance from a common storage facility, the fungi can spread to remote areas and could remain viable even when the external climatic conditions are unfavorable. The study suggests that humans themselves may represent a vast reservoir for these fungi as commensals, and humans could therefore play a significant role in their evolution and infection cycles.

Apart from the isolated fungi, the investigators also detected antifungal compounds on the surface of stored apples as well as on freshly picked apples from nonorganic orchards (1). This is not surprising because antifungals are commonly employed during storage to extend the life of fruit and during cultivation to reduce the risks of infection. More specifically, the authors detected azole drugs on fruit surfaces. Azoles are the most commonly used agricultural antifungal drugs, and their mode of action is similar to that of the azoles given in medical settings, i.e., targeting the ergosterol biosynthetic pathway. It is important to note that azoles are fungistatic and not fungicidal, meaning they inhibit growth but do not kill fungi. As a result, application of these compounds during apple storage might prime fungi to grow in the presence of such drugs, making them resistant to clinically relevant azoles because the mechanism of resistance is the same for both types of azoles. The cross-resistance of fungi to medical and agricultural antifungals has been demonstrated previously in multiple studies (6). Indeed, cross-resistance between medical and agricultural azoles was identified in most C. auris isolates in this study when they were tested for antifungal drug susceptibility. Importantly, isolates of C. auris are haploid in contrast to more prevalent pathogens, C. albicans and C. tropicalis, which are diploid. As a result, C. auris possesses more flexibility in terms of acquiring mutations faster in response to antifungal drugs and adapting to those drugs for survival. One possibility is that a few strains were able to tolerate and adapt to the presence of antifungals on the apple surface and only those were isolated in the study. This hypothesis is further supported by the observation that only a few yeast species that are known for antifungal drug resistance were isolated from apples. An interesting finding was the lack of C. albicans among fungal isolates from stored apples despite being the major pathogen in this clade. One possible explanation could be a lower prevalence of C. albicans in the area sampled. C. albicans isolates are less frequently antifungal drug-resistant than the species isolated, and this might have contributed further (7). This observation strengthens evidence that agricultural antifungals select and prime these fungal species for azole resistance. Thus, the usage of excessive antifungals in the field and during storage presents a dangerous situation for the development of drug resistance. The findings further suggest that drug-resistant, deadly fungal species may be selected through these routes and pose a significant challenge to human health.

The association of fungi with fruit is well established; however, isolation of commensal fungi from fruit is not well documented. This attempt to identify the ecological niche of successful human pathogens is crucial and should pave the way for further research. Isolation of C. auris from sources other than humans has so far been from India, a tropical country (4). Whether one would find C. auris on apples in temperate conditions will be interesting to explore, all the more so because the evolution of this fungus has been associated with increasing global temperature that might lead to adaptation of this fungus to grow at human body temperature (5). This feature combined with the ability to frequently develop antifungal drug resistance makes C. auris a formidable pathogen. Elucidating whether these features evolved simultaneously or sequentially in C. auris will also be important to fully understand the infection capacity of C. auris. Unfortunately, antifungal drug resistance is increasing, and cross-resistance between medical and agricultural antifungals is of significant concern. This may also contribute to the increasing prevalence of non-albicans *Candida* species. Looking ahead, it will be important to regulate the use of antifungal drugs, especially those that are fungistatic, in both medical and agricultural settings. In the lack of such measures, more antifungal resistance will arise, and it may result in pathogenic fungi evolving into more deadly variants and might further contribute to the evolution of nonpathogenic fungi into pathogenic ones.
